# Mr. Clement, on Apoplexy

**Published:** 1802-06

**Authors:** W. Clement

**Affiliations:** Apothecary. Shrewsbury


### To Dr. BRADLEY.

SIR,

HAVING no wish to enter into the Controversy between Dr. Langslow and Mr. Crowfoot on the Cause and Treatment of Apoplexy, I only beg leave to state the following case which occured to me a few months ago. Mr. S. B. aged 30, of a remarkably full habit of body, short neck, inactive, and much indisposed difpofed to any kind of exercife, was fuddenly feized on the 30th of September laft, immediately after dinner, with a moft violent head-ach, which foon deprived him of all fenfe and motion. He would have fallen to the ground, had he not been fupported by two ladies who were very fortunately with him at the time. On my arrival, (which was in a very few minutes) I found his extremities cold, countenance florid, refpiration difficult and intermitting, pulfe hard and full; and every fymptom indicated what *I considered* Apoplexy. I immediately took ten ounces of blood from the arm, at the fame time had his feet immerfed in warm water which was well faturated with common falt. I waited for full half an hour; but he did not appear in the leaft benefited by bleeding, all his fymptoms ftill continued unabated. With fome difficulty I got down an emetic, compofed of fifteen grains of white vitriol, one ounce of ipecacuanha wine, and twenty drops of the tincture of foxglove, which in the courfe of twelve minutes operated; the patient during the operation was fupported in an erect pofture. After the firft or fecond effort of vomiting he was relieved, became more fenfible, and exclaimed, O my head, my head! He gradually recovered during the operation of the emetic; and in the courfe of two or three hours was perfectly reftored, excepting a dull head-ach, which was likewife removed by a brifk cathartic that evening. I found him perfectly well the next morning; and he hath continued fo ever fince. This gentleman now lives more abftemioufly, takes more exercife, and avoids much fermented liquors. I do not prefume to make any comments on the above cafe, I ftate it fimply as it occurred, and what the refult was; but I may be permitted to fay, that *I believe* the emetic faved his life, and Dr. Langflow and others may confider it as a dangerous practice if they pleafe.

Permit me to return thanks to the authors of the many candid obfervations on Apoplexy, particularly to the ingenious and learned Pyrrho, likewife to Philo-Medicus for his valuable hints and interrogatories. Should the above cafe be confidered worth inferting in your very ufeful Journal, by giving it a place in your next Number, you will much oblige,

*Shrewsbury*, *May* 9, 1802. Your's, &c.

W. CLEMENT, Apothecary.

